# PEG 400-Based Phase Change Materials Nano-Enhanced with Functionalized Graphene Nanoplatelets

**DOI:** 10.3390/nano8010016

**Published:** 2017-12-29

**Authors:** Marco A. Marcos, David Cabaleiro, María J. G. Guimarey, María J. P. Comuñas, Laura Fedele, Josefa Fernández, Luis Lugo

**Affiliations:** 1 Departamento de Física Aplicada, Universidade de Vigo, 36310 Vigo, Spain; mmarcosm@uvigo.es (M.A.M.); luis.lugo@uvigo.es (L.L.); 2 Institute of Construction Technologies, National Research Council, 35127 Padova, Italy; laura.fedele@itc.cnr.it; 3 Laboratorio de Propiedades Termofísicas, Grupo NaFoMat, Departamento de Física Aplicada, Universidade de Santiago de Compostela, 15782 Santiago de Compostela, Spain; mariajesus.guimarey@usc.es (M.J.G.G.); mariajp.comunas@usc.es (M.J.P.C.); josefa.fernandez@usc.es (J.F.)

**Keywords:** graphene nanoplatelets, poly(ethylene glycol), NePCM, solid-liquid phase change, thermal conductivity, dynamic viscosity, volumetric behaviour

## Abstract

This study presents new Nano-enhanced Phase Change Materials, NePCMs, formulated as dispersions of functionalized graphene nanoplatelets in a poly(ethylene glycol) with a mass-average molecular mass of 400 g·mol^−1^ for possible use in Thermal Energy Storage. Morphology, functionalization, purity, molecular mass and thermal stability of the graphene nanomaterial and/or the poly(ethylene glycol) were characterized. Design parameters of NePCMs were defined on the basis of a temporal stability study of nanoplatelet dispersions using dynamic light scattering. Influence of graphene loading on solid-liquid phase change transition temperature, latent heat of fusion, isobaric heat capacity, thermal conductivity, density, isobaric thermal expansivity, thermal diffusivity and dynamic viscosity were also investigated for designed dispersions. Graphene nanoplatelet loading leads to thermal conductivity enhancements up to 23% while the crystallization temperature reduces up to in 4 K. Finally, the heat storage capacities of base fluid and new designed NePCMs were examined by means of the thermophysical properties through Stefan and Rayleigh numbers. Functionalized graphene nanoplatelets leads to a slight increase in the Stefan number.

## 1. Introduction

Thermal energy storage (TES) is considered one of the key technologies for the energy production of the future, especially in the case of renewable systems for which the intermittency and less predictable nature of energy sources are major issues [1,2]. Among different types of heat storage, it is worth mentioning the higher densities of energy storage and almost isothermal characteristics of those storage processes that take advantage of the energy involved in a phase change, in comparison to those methods which simply utilize the sensible heat due to a temperature difference [3,4]. Those materials that use latent heat to store and release energy, increasing thermal inertia of those systems in which are integrated, are also known as phase change materials, PCMs [4,5]. Over the last decades, many different groups of materials have been proposed as PCMs, like inorganic compounds (salt and salt hydrates), organic compounds (paraffins, alcohols, fatty acids and polymers), or eutectics [2,3]. In particular, polymeric materials such as poly(ethylene glycols), PEGs, have been extensively studied as potential solid-liquid PCMs due to their promising characteristics such as appropriate phase change temperature and enthalpy, which can be tuned through the molecular mass, congruent melting behaviour, non-toxicity, low vapour pressure and competitive price, among others [2,5,6]. However, as many other organic phase change materials, PEGs exhibit low thermal conductivities which prolongs storing and releasing process and limits their usage in practice [5,7]. In order to overcome this limitation and to develop high-performance phase change materials, the dispersion of nano-sized materials with high thermal conductivity has gained increasing attention, given rise to what is known as Nano-enhanced Phase Change Materials, NePCMs, or nano-PCMs [8,9].

Owing to their properties, poly(ethylene glycols), which are a type of polyglycols or polyols, are among the most adaptable chemical ingredients and processing aids available to formulators and manufacturers of a wide range of products. In particular, special attention must also be paid to their potential use as hydraulic fluids and lubricants since poly(alkylene glycols) are considered to exhibit excellent lubricating ability for gears and compressors, for example [10,11]. Cooling and decreasing friction between moving parts, preventing corrosion and sealing and cleaning engines are among lubricant main functions. Thus, the development of new advanced lubricants that allow reducing friction between mechanical elements in motion is a key issue to improve machinery efficiency and component durability and, in turn minimize harmful gas emissions to the environment. In this sense, one of the used, current and promising strategies to improve performance of machinery lubrication and coolant applications is the use of nanoadditives [12,13].

Regarding colloidal dispersions using PEGs as carrier fluids, SiO_2_ is the most studied additive in the development of new phase change materials with improved thermal properties [6,14,15,16,17]. Tang et al. [15] prepared form-stabilized SiO_2_/PEG composites with latent heats over 100 J·g^−1^ using polymeric chains with mass-average molecular masses ranging from 1000 to 6000 g·mol^−1^ as base fluids. Min et al. [14], Quian et al. [6] and Wang et al. [17] also utilized SiO_2_ to increase thermal conductivity and form-stabilized PEG 4000 in the two first studies and PEG 10000 in the latter. Wang et al. [18] formulated new composite PCMs from a SiO_2_ gel and a PEG 1000 solution at 15/85 ratio doped with β-aluminium nitride at concentrations ranging from 5% to 30% and obtained remarkable enhancements in thermal conductivity, up to 157% higher than the pure poly(ethylene glycol), with suitable latent heats of fusion and slight modifications of melting and crystallization temperatures. Tang and co-workers studied SiO_2_/PEG 6000 composite PCMs doped in situ with Cu [19], Al_2_O_3_ [7] and Ti_4_O_7_ [20] and reported thermal conductivity improvements compared to the SiO_2_/PEG 6000 matrix of 14%, 11% and 73.1% with 2.1% mass concentration of copper, 3.3 wt % Al_2_O_3_ loading and 3 wt % Ti_4_O_7_ concentration, respectively. A reduction in subcooling of about 7 K was found for Cu-doped NePCMs compared to pure PEG 6000 [19]. Zafarani-Moattar et al. [21,22] analysed the thermophysical properties and rheological behaviour of ZnO dispersions in PEG 400, while Zendehasbagh et al. [22] prepared NiO nanofluids based on poly(ethylene glycols) mixtures of PEG 400 with PEG 2000 or PEG 6000.

In recent years carbon allotropes have risen great interest in the preparation of nanostructured materials due to their unique physical properties and its relative ease and cost of synthesis on a large scale [23,24,25,26]. Thus, certain carbon-based nanostructures exhibit thermal conductivities much higher than metallic or metal oxide nanoparticles [27]. Regarding PCMs, expanded carbon based microstructures or nanostructures have proven to be remarkably effective as thermal conductivity enhancers and shaper stabilizers of different phase change materials such as paraffins [28,29,30,31], fatty acids [32,33,34,35], or polymers [25,36,37,38,39,40,41,42], with acceptable reductions in latent storage heat. In the case of poly(ethylene glycols), Wang et al. [36] reported reductions in melting temperature of PEG 6000—based NePCMs with the addition of different surface-functionalized graphene oxides. Authors attributed these diminutions, especially noticeable with the presence of carboxylic groups, to the strong interactions created between nanomaterial surfaces and polymeric chains. Wang et al. [37] presented a novel solid-solid phase change material also formulated as functionalized graphene oxide (GO) dispersions in poly(ethylene glycol) 6000. Qi et al. [25] prepared a novel form-stable nanocomposite by simple blending and impregnation method with PEG 6000 as thermal storage material and graphene oxide as supporting substance. Authors reported melting temperatures quite similar at different composite ratios, while crystallization temperatures did not show a clear loading of graphene oxide dependence. Otherwise, Wang et al. [40] used a dynamic impregnating absorption method to design a nanocomposite based on microporous carbon supporting matrix using PEG 4000. Their new proposal materials show noticeable thermal conductivity enhancements in relation to the pure PEG. Zhang et al. [39] prepared novel white carbon black/PEG 4000 form-stable composite PCMs by super ultrasound assisted. Thermal conductivity of these materials presented improvements up to 50% in relation to those of pure PEG.

Zhang et al. [38] analysed the thermal conductivity of poly(ethylene glycol) phase change materials nano-enhanced with carbon coated with Cu, Al and Fe nanoparticles and found 49%, 40% and 30% enhancements in thermal conductivity for 1.5 wt % nanoadditive loadings, respectively. Hybrid NePCMs were also designed by Liu et al. [41] and Tang et al. [42] consisting of SiO_2_/PEG 6000 matrices doped with carbon fibres in the former case and with Multiwall Carbon Nanotubes, MWCNTs, in the later. Carbon nanostructures are also adequate solid nanoadditives to promote new nanostructured lubricants [13]. Thus, Gupta et al. [13] have recently studied the effect of the concentration of reduced graphene oxide in poly(ethylene glycol) PEG 200 on the decrease of wear and coefficient of friction on steel surfaces lubricated with this type of dispersions.

In this paper, new nano-enhanced phase change materials and nanolubricants designed as dispersions of functionalized graphene nanoplatelets (fGnP) in a poly(ethylene glycol) with a mass-average molecular mass of 400 g·mol^−1^, PEG 400, were developed. Specifically, it was studied four different mass concentrations of (0.05, 0.10, 0.25 and 0.50) wt %, which correspond to (0.0003, 0.0005, 0.0013 and 0.0025) volume fractions, respectively, considering a density value of 2.25 g·cm^−3^ for the fGnP nanopowder [43]. In order to analyse thermal energy storage capabilities, the influences of nanoadditive loading on the thermophysical properties of density, dynamic viscosity, thermal conductivity and heat capacity were experimentally studied in a wide temperature range. In addition, temperatures and enthalpy changes associated with (solid-liquid) phase change transitions were also investigated for the designed dispersions.

## 2. Materials and Methods

### 2.1. Materials

Functionalized graphene nanoplatelets, fGnP, were provided by Iolitec with declared mass purity of 99.5% and average thickness of 11–15 nm. fGnP nanopowder was used as supplied, no further chemical treatment of nanoplatelet surface or surfactant addition was taken into consideration in this work. A pharmaceutical-grade poly(ethylene glycol) PEG 400 was purchased from Panreac AppliChem (Barcelona, Spain). A Mettler AE-200 analytical balance (Mettler Toledo, Greifensee, Switzerland) with an accuracy of 1 × 10^−5^ g was used to weigh reagents and samples.

### 2.2. Graphene and Poly(Ethylene Glycol) Characterization

**Figure 1:**
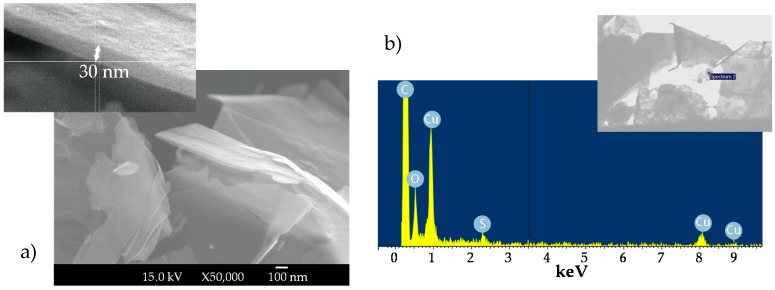
(**a**) SEM images at two magnifications: ×50,000 and ×300,000 (insertion) and (**b**) EDS microanalysis of fGnP powder.

Morphology and size of graphene nanostructure were observed under a scanning electron microscope (SEM) JEOL JSM-6700F (JEOL, Tokyo, Japan) operating at an accelerator voltage of 20 kV in backscattering electron image (YAG type detector). In order to prepare SEM samples, a drop of graphene in methanol was deposited on the top of a formvar-covered copper transmission electron microscopy grid and then the solvent was evaporated at atmospheric conditions. Typical SEM images of the graphene nanopowders are presented in Figure 1a. 

In a first inspection, it is observed that in the commercial powder graphene is stacked forming domains of up to several micrometres. By studying the specimen in more detail, it is possible to differentiate several nanometre-thick laminar structures of rough surfaces, which correspond with the lamellar morphology previously reported for graphene platelets [44,45]. SEM technique was also combined with energy dispersive X-ray spectroscopy (EDS Oxford Inca Energy 300 SEM, Oxford Instruments, Abingdon, UK) in order to analyse the elemental composition of the dry graphene nanopowder. Figure 1b shows an example of EDS microanalysis and, as expected, results indicate the predominant presence of carbon and oxygen in the sample. Marginal contents of other elements such as sodium, calcium, or sulphur were also detected; their presence in the nanopowder can be due to the route used to synthesize the nanomaterial.

**Figure 2:**
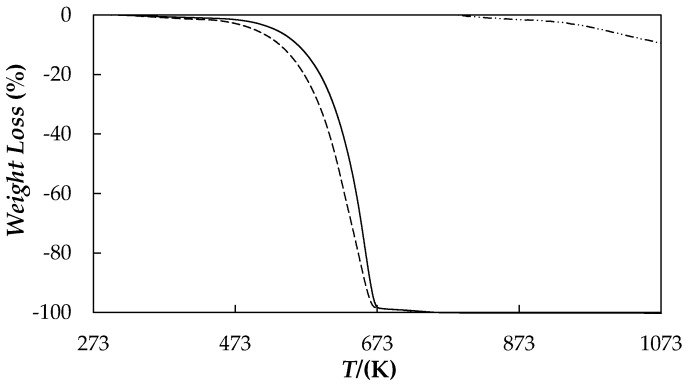
TGA curves of: PEG 400 (— —), fGnP nanopowder (– ·· –) and 0.5 wt % fGnP/PEG 400 NePCM (**―**) under nitrogen atmosphere.

Thermal stabilities of as received nanopowder and base material were studied using a TGA/DSC1 thermogravimetric analyser (Mettler-Toledo GmbH, Greifensee, Switzerland). About 25 mg of sample was subject to temperature sweeps from ambient temperature to 1073 K at a heating rate of 10 K/min and under an inert nitrogen atmosphere. Figure 2 shows TGA profiles of graphene nanoplatelets and poly(ethylene glycol).

A gradual weight loss was found for functionalized graphene nanoplatelets as temperature increases, with a total weight loss lower than 9.5% for the entire analysed temperature range. However, when air is introduced into the chamber at the highest temperature the nanopowder sample was almost completely calcined, with an additional weight loss of about 82%. For the PEG 400 used as base material, the weight loss step occurs mainly at temperatures between 460 K and 670 K, with a weight loss less than 5% for temperatures below 503 K and a *T_onset_* degradation temperature of about 507 K. *T_onset_* temperatures were obtained as the temperature of the crossing point between thermogram baseline and the tangent line to derivative weight loss curve at the inflection point.

**Figure 3:**
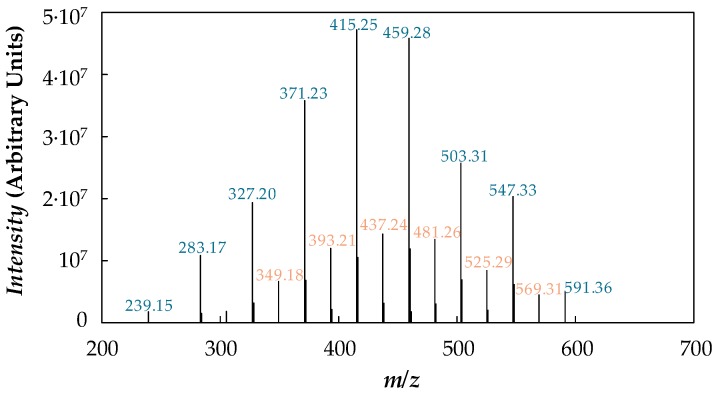
Electrospray mass spectrum of PEG 400.

Purity and molecular mass of the poly(ethylene glycol) were analysed by electrospray ionization mass spectrometry (ESI-MS) on an APEXQe FT-ICR MS mass spectrometer (Bruker Daltonics, Billerica, MA, USA) equipped with a 7 T actively shielded magnet. A Combi MALDI-electrospray ionization (ESI) source was used to produce ionization with a voltage of 4.5 kV applied to the needle and a counter voltage of 300 V applied to the capillary. A PEG sample was dissolved in a spray solution of CH_3_OH/H_2_O/formic acid (70:29.9:0.1 by volume). Data were acquired using the ApexControl software version 3.0.0 and the spectrum was processed using the DataAnalysis software version 4.0, both software from Bruker Daltonics. Figure 3 reports the ESI mass spectrum of the PEG 400 used as base material.

Taking into account the molecular structure of the polymer, HO–[CH_2_–CH_2_–O]_n_–H, all peaks present in the mass spectrum are attributable to PEG molecules cationized with H^+^ (*m*/*z* = 19.02 + 44.03 *n* with *n* = 5–13) or Na^+^ (*m*/*z* = 41 + 44.03 *n* with *n* = 7–12). Hence, the degree of impurities in the specimen can be considered negligible. The distribution of the molecular mass is uniform with a number average of *M_n_* = 417.4 g·mol^−1^, an average value *M_w_* = *∑N_i_·M_i_*^2^*/∑N_i_·Mi* of 427.2 g·mol^−1^ where *N_i_* is the number of moles of species i, and a polydispersity index, *M_w_*/*M_n_* = 1.023, which indicates that the polymer is quasi-monodisperse.

Water content in PEG 400, which was determined using a Metrohm 870 Karl Fischer KF Titrino (Metrohm, Herisau, Switzerland) with a Titran 2 (Merck, Darmstadt, Germany), is less than 300 ppm of water.

### 2.3. NePCM Preparation, Stability and Chemical Interactivity Analysis

**Figure 4:**
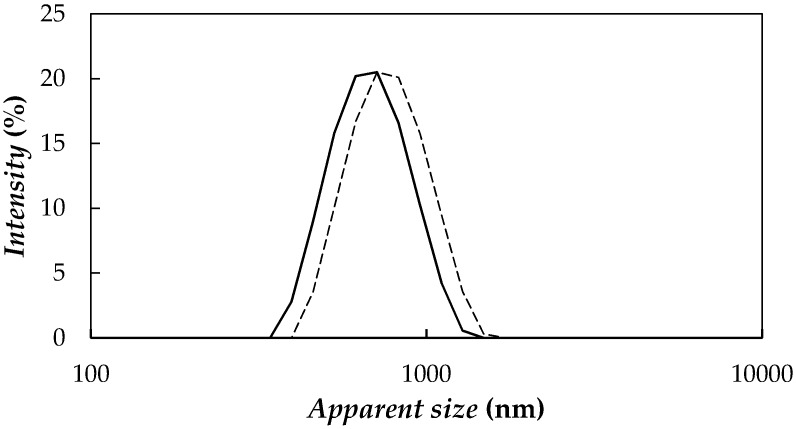
Apparent size distributions of 0.25 wt % fGnP/PEG 400 dispersions prepared by using ultrasonic bath for 240 min (**―**) and ultrasonic probe for 70 min (——).

NePCMs were prepared following a two-step method combining mechanical stirring with a vortex mixer and ultrasonication. Two different ultrasonic devices were considered: (i) an Ultrasounds (JP Selecta SA, Barcelona, Spain) 9 L ultrasonic bath at 20 kHz and power of 200 W and (ii) a Bandelin Sonopuls HD2200 (Bandelin, Berlin, Germany) ultrasonic disruptor, with a maximum power output of 200 W and a frequency of 20 kHz, working with a 3 mm diameter titanium microtip at a fixed 20% amplitude. In order to select the best conditions for the preparation of the graphene nanofluids based on PEG 400, a study of the sonication time influence on the apparent nanoparticle size distributions of dispersions prepared with these two sonication devices was carried out by using a Zetasizer Nano ZS (Malvern Instruments, Malvern, UK) based on Dynamic Light Scattering, DLS, technique. It must be mention that size distributions obtained from the random changes in the intensity of light scattered using DLS technique are based on the assumption that particles are spherical while studied nanoadditives are sheet-like shaped. Measurements were carried out at 298.15 K using a scattering angle of 173°. Figure 4 shows size distribution of 0.25 wt % fGnP/PEG 400 dispersions prepared using both methods, as an example. These results are similar to those obtained for the rest of the concentrations. A reduction in average apparent size of nanoparticle aggregates was observed by increasing sonication time, minimum values being 881 nm and 700 nm for 240 min of ultrasonication bath and 45 min of disruptor, respectively. Thus, the last sonication device was considered more appropriated to PEG 400-based fluids. In addition, although no reduction in size measurements was observed between 45 min and 70 min of ultrasonication with the disruptor, 70 min sample exhibits slightly better temporal stability. Therefore, the ultrasonic probe and 70 min sonication time were selected to prepare all the studied fGnP/PEG NePCMs.

Thermal stability of 0.5 wt % fGnP/PEG 400 dispersion was also analysed in the same conditions as base materials by thermo-gravimetric analysis and results were incorporated in Figure 2. The weight loss profile of the NePCM is very similar to that of PEG 400, which indicates that the addition of the nanoadditive does not lead to any PEG transformation. However, a slight improvement in thermal stability, with a displacement in weight loss-temperature curve of 18 K to the right for the studied NePCM, is observed with the addition of the graphene. This result is attributed to the better thermal stability of nanopowder, higher than 1000 K, in comparison to the poly(ethylene glycol). Similar results were also reported in previous studies [46,47].

**Figure 5:**
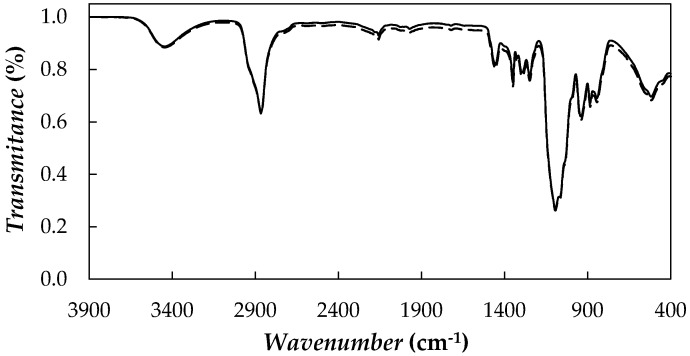
FT-IR spectrum of PEG 400 (——) and 0.5 wt % fGnP/PEG 400 (**―**).

In order to analyse possible new chemical interactions between the base fluid and nanoadditives, Fourier Transform Infrared (FT-IR) spectra were recorded for the PEG 400 and 0.5 wt % NePCM in the wavenumber range from 400 to 4000 cm^−1^ by using FTIR spectroscopy (Varian 670-IR with PIKE GladiATR accessory, Agilent Technologies, Santa Clara, CA, USA). FT-IR spectra of the two samples are shown in Figure 5.

In the case of pure PEG 400, typical bands of these polymers [7,19,48] can be observed at wavenumbers of 3446 (O–H stretching), 2865 (–CH_3_ stretching) and 1094 cm^−1^ (C–O–C symmetrical stretching). Tang et al. [7,19] observed the same bands when performing FT-IR measurements for a PEG with a molecular mass of 6000 g·mol^−1^. In that case, the bands were shown at 3440, 2917, 1106 cm^−1^ [7] or 3430, 2917 and 1106 cm^−1^ [19]. No new spectral peaks or shifts are found for the dispersions prepared at the highest nanoparticle loading, which would indicate that no chemical bonds between the poly(ethylene glycol) and graphene were formed in the NePCMs.

### 2.4. Thermophysical Characterization

(Solid-liquid) phase transitions of poly(ethylene glycol) and nano-enhanced phase change materials were experimentally obtained in the temperature range from 188 to 313 K using a differential scanning calorimeter, DSC, Q2000 (TA Instruments, New Castle, DE, USA). The instrument is equipped with a refrigerated cooling system RSC90 and nitrogen with mole fraction purity higher than 0.99999 was used as purge gas. General calibration of the device was carried out according to manufacturer procedure. Approximately 10 μg of sample was hermetically encapsulated in 20 μL Tzero aluminium pans able to withstand pressures up to 0.4 MPa. Pans were weighed before and after tests to verify that no loss of mass had taken place and therefore validate measurements. Before experiments, samples were heated up at a temperature of 313 K, at least 20 K above their melting point and held for 10 min to remove any prior thermal history of the materials. Data analysis was performed using Universal Analysis 2000 software V4.5A (TA Instruments, New Castle, DE, USA). Estimated uncertainties for transition temperatures and enthalpies are 0.3 K (with a repeatability of 0.1 K) and 1.2 J·g^−1^ (with a repeatability of 0.7 J·g^−1^), respectively [49].

Isobaric heat capacities, *C_p_*, of PEG 400 and graphene nanoplatelet powder were measured between 233 and 373 K employing the differential scanning calorimeter DSC Q2000. Measurements were performed using a quasi-isothermal Temperature-Modulated Differential Scanning Calorimetry (TMDSC) method by which the sample temperature sinusoidally varies with amplitude of 0.5 K and oscillation period of 80 s for at least 40 min. Estimated experimental uncertainty for this property in the studied temperature range is 3% [50].

Thermal conductivities, *k*, were measured in liquid phase and at temperatures ranging from 283 to 333 K by means of a KD2 Pro Thermal Properties Analyser (Decagon Devices, Inc., Pullman, WA, USA). This device was used together with a KS-1 sensor made of stainless steel with a length of 60 mm and a diameter of 1.3 mm and which is appropriate for measuring thermal conductivities of liquids between 0.020 and 4 W·m^−1^·K^−1^. In order to remove thermal gradients and ensure a uniform initial temperature, samples were fully immersed in a Grant GP200 (Grant Instruments, Cambridge, UK) oil bath and a delay of at least 15 min was waited between consecutive measurements. The estimated expanded uncertainty of this instrument is lower than 10 mW·m^−1^·K^−1^ for thermal conductivities below 200 mW·m^−1^·K^−1^ [51].

Dynamic viscosity, *ƞ* and density, *ρ*, were experimentally obtained at temperatures between 283 K and 373 K with a SVM 3000 rotational Stabinger viscometer-densimeter (Anton Paar, Graz, Austria). Density determinations are based on the well-known vibrating tube technique, whereas the cell for viscosity measurements is based on a modification of Couette principle. Schematic set up and basic operation principles are described in a European Patent, EP 0 926 481 A2. Viscosity values can be traced back to a single rotational speed, which is measured by an electronic system (Hall effect sensor) that counts the frequency of the rotating magnetic field. Kinematic viscosities fulfil repeatability, reproducibility and comparability requirements of ASTM D445. Expanded uncertainties of 0.02 K in temperature, 1% in viscosity and 0.05% in density were estimated [52].

## 3. Results

### 3.1. Phase Change Characterization

**Figure 6:**
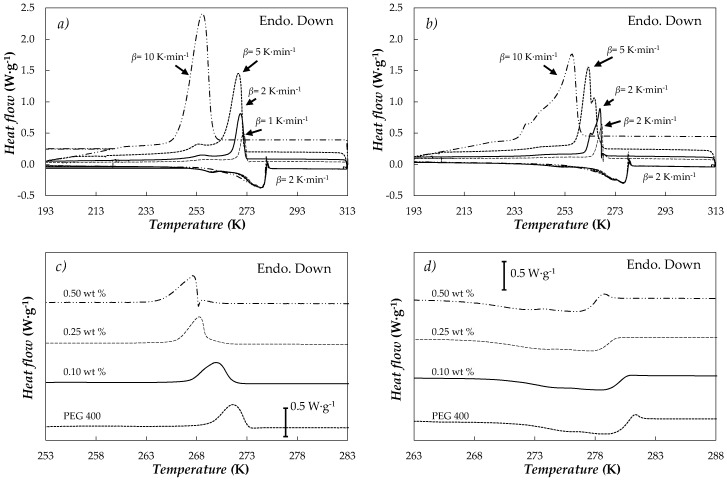
DSC thermograms of (**a**) PEG 400 and (**b**) 0.5 wt % fGnP/PEG 400 concentration at different cooling rates, *β* and a heating rate of *β* = 2 K·min^−1^. (**c**) Cooling and (**d**) heating scans of the different NePCMs at *β* = 1 K·min^−1^ rates.

(Solid-liquid) transitions of the PEG 400 used as base material and three NePCMs (0.10, 0.25, 0.50) wt % were analysed by means of temperature sweeps with cooling rates ranging from 1 to 10 K·min^−1^ and heating rates of 1 and 2 K·min^−1^. After the cycles necessary to analyse the samples at the different cooling/heating rates, an additional DSC scan was performed using the same conditions of the first DSC run in order to check that there was not any difference in the (solid-liquid) phase characteristics and therefore validate the results. In addition, it must be pointed out that no significant differences with cycling were observed, specially checked for unmodified PEG 400 and highest mass concentration after 50 melting-freezing processes at 10 K·min^−1^. Figure 6a,b shows the thermograms obtained for PEG 400 and 0.5 wt % dispersion of nanoplatelets at different cooling rates, 2 K·min^−1^ being the heating rate.

Recrystallization temperature of PEG 400 decreases with the increasing cooling rate. This same behaviour was also observed by Pielichowski et al. [53] for poly(ethylene glycols) consisting of longer polymeric chains with molecular masses between (1000 and 35,000) g·mol^−1^. This phenomenon is attributed to the different thicknesses of the formed crystals during the solidification of this type of materials because of the multiple folds of the polymer chains [53,54]. The behaviour for 0.5% concentration is also exhibited by the other analysed NePCMs. Figure 6c,d present crystallization and melting curves of PEG 400 and studied NePCMs obtained at 1 K·min^−1^ rates. Crystallization and melting temperatures and melting heats obtained at those conditions are gathered in Table 1.

Within the studied concentration range, graphene addition reduces crystallization temperature by up to 4 K. Similarly, the temperature range in which the melting transition takes place also decreases in 2.5 K with the loading of nanopowder. Wang et al. [36,47] found the same effects when they studied graphene oxide and reduced graphene oxides nanocomposites in poly(ethylene glycol) PEG 6000. These authors attributed this behaviour to a possible reduction in the movement of the poly(ethylene glycol) segments during melting and crystallization processes due to the presence of nanoadditives. Functionalized graphene nanoplatelets used as nanoadditive are not an active PCM in the studied temperature range. Therefore, a reduction in latent heat of fusion is expected as fGnP loading increases. However, the lower sample fraction which undergoes melting transition due to fGnP addition cannot by itself explain the observed diminutions in *ΔH_m_*. This further reduction was attributed to a diminution in poly(ethylene glycol) crystallinity as a consequence of nanomaterial dispersion in the literature [36,47]. This effect can be characterized by using the degree of crystallinity, *X_c_*:

(1) $$X_{c}=\frac{\Delta H_{m}}{\left(1-\phi \right)\cdot \Delta H_{m,\mathrm{pure}}}$$

where *ΔH*_m_ and *ΔH*_m,pure_ are the melting heats of sample and pure PEG 400, respectively and *ϕ* is nanoplatelet mass fraction. In this case, a reduction in degree of crystallinity from 90% to 83% was observed with, considering *ΔH*_m,pure_ = 117.6 J·g^−1^ [2].

### 3.2. Isobaric Heat Capacity

**Figure 7:**
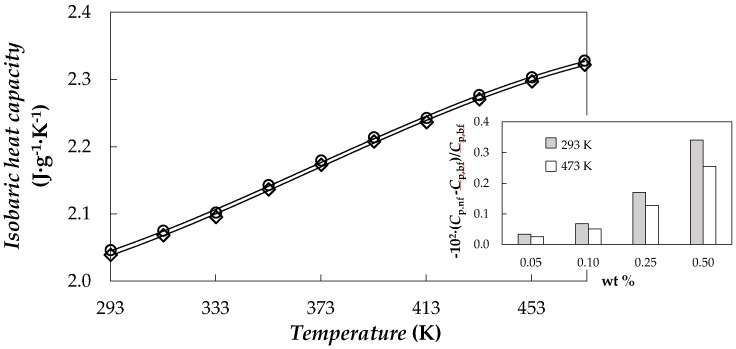
Temperature dependence of isobaric heat capacity, *C_p_*, of PEG 400 and 0.5 wt % NePCM. Inset: isobaric heat capacity decreases, −10^2^·(*C_p_*_,nf_ − *C_p_*_,bf_)/*C_p_*_,bf_, vs. mass concentration, wt %, at different temperatures.

Isobaric heat capacity was experimentally determined for the base fluid and nanopowder. Obtained values for PEG 400 show an average absolute deviation, AAD%, of 3% with respect to those data published by Francesconi et al. [55]. Nanopowder *C_p_* values range from 0.44 to 1.14 J·K^−1^·g^−1^ within the studied temperature range, which is in agreement with the values reported by Pop et al. [43]. For NePCMs, *C_p_* values were obtained using the following equation [56,57].

(2) $$C_{p,\mathrm{nf}}=\varphi \cdot C_{p,\mathrm{np}}+\left(1-\varphi \right)\cdot C_{p,\mathrm{bf}}$$

where *ϕ* is the mass fraction of nanoplatelets and nf, np and bf subscripts correspond to nanofluid, nanopowder and base fluid, respectively. Figure 7 presents the temperature dependence of *C_p_* values for base fluid and the highest studied concentrations together with modifications of NePCM isobaric heat capacities regarding the PEG 400 at different temperatures. The obtained *C_p_* values decrease slightly with the concentration of functionalized graphene nanoplatelets up to 0.34% for the highest analysed concentration, the 0.5 wt % NePCM. These reductions are lower as temperature rises.

### 3.3. Thermal Conductivity

**Figure 8:**
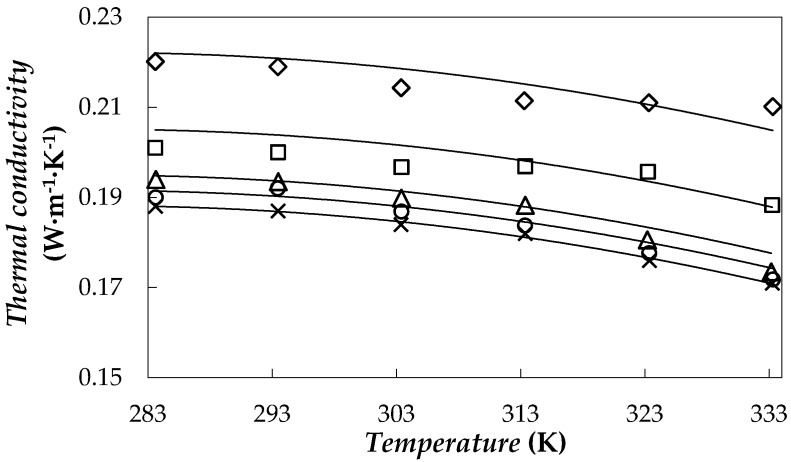
Thermal conductivity against temperature for PEG 400 (╳) and for 0.05% (○), 0.10% (△), 0.25% (□) and 0.50% (◇) fGnP/PEG 400 nanofluids. Nan et al. equation [58] (**―**).

The influence of the fGnP loading on thermal conductivity was analysed for (0.05, 0.10, 0.25 and 0.50)% mass concentrations. Figure 8 shows thermal conductivity behaviour in relation to temperature for the base fluid and studied NePCMs.

Experimental tests show that the thermal conductivity increases with the loading of fGnPs, with enhancements up to 23% for the highest concentration. In order to describe the effect of dispersed nanoparticles on thermal conductivity, Nan et al. model [58] was used:

(3) $$k_{\mathrm{nf}}=k_{\mathrm{bf}}\cdot \frac{3+\phi \cdot \left[2\cdot \beta_{11}\cdot \left(1-L_{11}\right)+\beta_{33}\cdot \left(1-L_{33}\right)\right]}{3-\phi \cdot \left(2\cdot \beta_{11}\cdot L_{11}+\cdot \beta_{33}\cdot L_{33}\right)}$$

where *L*_ii_ are geometric parameters that take values of *L*_11_ = 0 and *L*_33_ = 1 in the case of graphene lamellar nanostructures such as that used in this work [59,60], *φ* is the volumetric fraction of nanoplatelets and *β*_ii_ is a coefficient defined as:

(4) $$\beta_{\mathrm{ii}}=\frac{k_{\mathrm{np}}-k_{\mathrm{bf}}}{k_{\mathrm{bf}}+L_{\mathrm{ii}}\cdot \left(k_{\mathrm{np}}-k_{\mathrm{bf}}\right)}$$

In this study, the Nan equation was used as a correlation with the in-plane thermal conductivity of the graphene, *k*_np_, as fitting parameter. A description of the thermal conductivities of the whole system with an AAD% between experimental and correlated data of 1% was obtained with *k*_np_ = 20 W·m^−1^·K^−1^. This result of in-plane thermal conductivity is slightly higher than the values of 11 and 17 W·m^−1^·K^−1^ obtained by Kole and Dey [59] or Cabaleiro et al. [60], respectively, when they studied graphene oxide dispersions in water-ethylene glycol mixtures. 

### 3.4. Volumetric Behaviour

**Figure 9:**
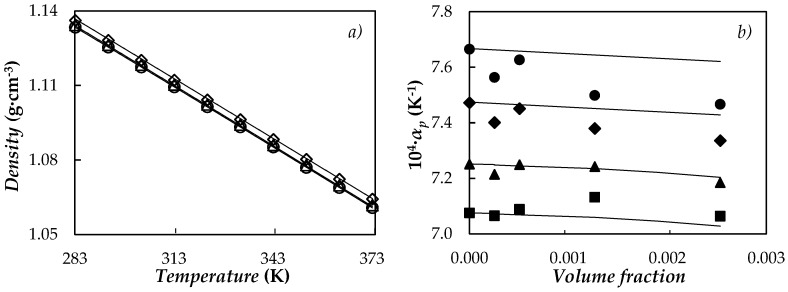
(**a**) Temperature dependence of density at different mass fractions: PEG 400 (○), 0.10% (△) and 0.5% (◇). (**―**) Equation (5). (**b**) Isobaric thermal expansivity, *α_p_*, vs. volume fraction at different temperatures: 288.15 K (■), 313.15 K (▲), 343.15 K (◆) and 368.15 K (●). (**―**) Equation (6).

Density was experimentally obtained for PEG 400 and four fGnP/PEG 400 nanofluids. A comparison between our density values for the base fluid and those previously reported in the literature for poly(ethylene glycols) with molar masses ranging from 365 to 414 g·mol^−1^ [55,61,62,63,64], shows average deviations below 0.1%. Temperature behaviour of density is plotted for base fluid and 0.10 and 0.50 wt % NePCMs in Figure 9a.

NePCM density increases with the concentration of nanoadditives as usual in nanostructured thermal materials. The temperature does not practically influence these density increments for temperatures below 333.15 K. However, density improvements rise with temperature at values higher than 333.15 K, ranging from 0.25% to 0.33% for 0.5 wt % fGnP/PEG 400 nanofluid, as an example. The isobaric thermal expansivities, *α_p_* = −(1/*ρ*)(∂*ρ*/∂*T*)*_p_*, were numerically calculated from the derivatives of the polynomial density adjustments. As can be seen in Figure 9b, *α_p_* increases with temperature up to 9% in the studied range while this coefficient decreases with the concentration of nanoplatelets, up to 2.6% in the case of 0.5 wt % fGnP/PEG 400 nanofluid at 368 K.

Densities and isobaric thermal expansivities here reported for NePCMs were also compared with values predicted by the following equations:

(5) $$\rho_{\mathrm{nf}}=\phi \cdot \rho_{\mathrm{np}}+\left(1-\phi \right)\cdot \rho_{\mathrm{bf}}$$

(6) $$\alpha_{p,\mathrm{nf}}=\phi \cdot \alpha_{p,\mathrm{np}}+\left(1-\phi \right)\cdot \alpha_{p,\mathrm{fb}}$$

where *φ* is the volume fraction of the nanoplatelets, while the values of *ρ*_np_ and *α_p,_*_np_ were obtained from literature [43,65]. The maximum deviations obtained between the experimental values and data from Equations (5) and (6) are less than 0.1% for density and 2.4% for isobaric thermal expansivity.

### 3.5. Thermal Diffusivity

In non-steady state or transient conditions, the ability of materials to transfer thermal energy is described by thermal diffusivity, α, which relates thermal conductivity and volumetric heat capacity:

(7) $$\alpha =\frac{k}{\rho \cdot C_{p}}$$

**Figure 10:**
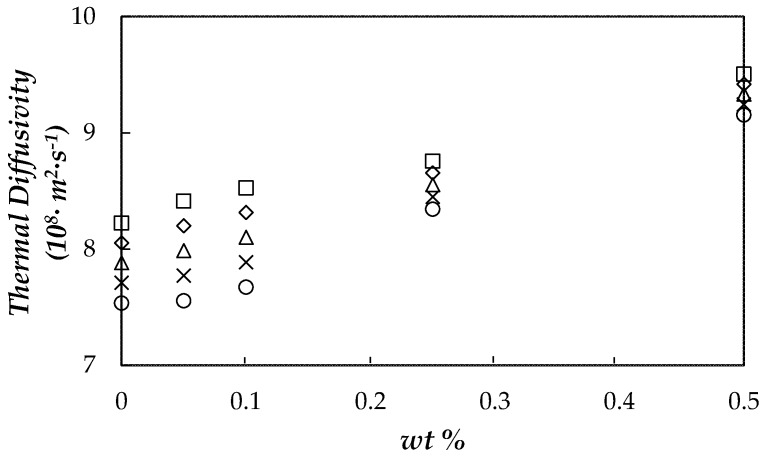
Mass fraction, wt %, dependence of thermal diffusivity at different temperatures: 293.15 K (□), 303.15 K (◇), 313.15 K (△), 323.15 K (╳) and 333.15 K (○).

Materials with high *α* exhibit fast responses to thermal changes in the environment and thus are preferred when it comes to transferring stored heat. In this work, thermal diffusivity was obtained for the PEG 400 and NePCMs from our experimental *k*, *ρ* and *C_p_* data. In order to calculate thermal conductivities and isobaric heat capacities at the exact temperature values, the temperature dependence of these two properties was first described by using a linear correlation in the case of *k* and a second-order polynomial fitting for *C_p_*. Obtained thermal diffusivities for PEG 400 and NePCMs are plotted in Figure 10.

Thermal diffusivity rises with nanoparticle loading and the decrease in temperature. The best results in terms of thermal diffusivity are obtained for 0.5 wt % NePCM, with a maximum enhancement compared to the base material of 21% at 333.15 K. Obtained maximum increases are lower than those reported by Babapoor et al. [66] for paraffin nanoparticle composite phase change materials. However, it should be taken into account that they used loadings of up to 8 wt % of metallic oxides as nanoadditives. Moreover, absolute thermal diffusivities of fGnPs/PEG400 are twice superior than those of those paraffin nanoparticle composites [66]. Lower absolute thermal diffusivities than these here reported were also reported for expanded graphite/stearic acid and carbon nanotubes/stearic acid NePCMs by Cheng et al. [67]. Finally, results in thermal diffusivity also show a potential use for the proposed NePCMs in distinctive thermal management applications.

### 3.6. Viscosity

**Figure 11:**
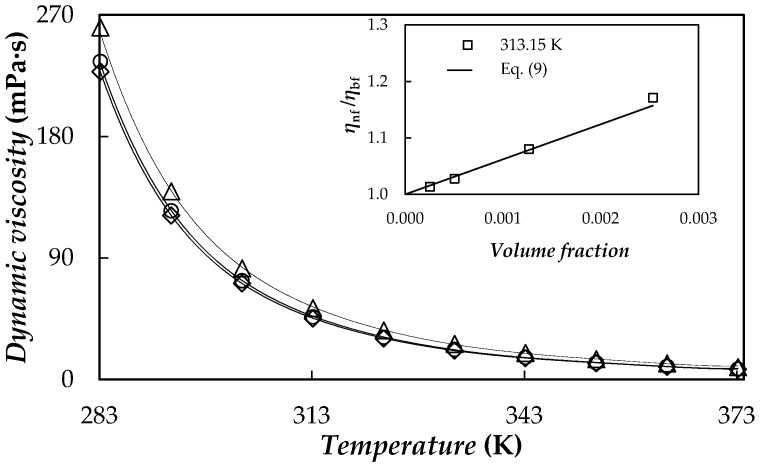
Temperature dependence of dynamic viscosity, *ƞ*, for: PEG 400 (◇), 0.10% (○) and 0.50% (△) mass concentrations. VFT equation (**―**). Inset: viscosity ratios, *η*_nf_/*η*_bf_, vs. nanoadditive volume fraction, together with the values provided by using Equation (9) with *φ*_m_ = 0.032.

Values obtained for PEG 400 present a good agreement with viscosity data reported for poly(ethylene glycols) with mass-average molecular masses ranging from 365 to 400 g·mol^−1^ [55,62,63,68]. An average deviation of 1.9% is obtained. Figure 11 shows the temperature dependence of the dynamic viscosity for the base fluid and two NePCMs.

As expected, dynamic viscosity decreases considerably with temperature. This behaviour can be described using the Vogel-Fulcher-Tamman equation (VFT):

(8) $$\ln\;\eta \left(T\right)=\ln\;\eta_{0}+\frac{D\cdot T_{0}}{T-T_{0}}$$

where *ƞ*_0_, *D* and *T*_0_ are the setting parameters. AADs% between experimental values and those from Equation (8) are equal to or less than 1.2%.

In order to show the effectiveness of the fit, the values obtained by using Equation (8) are also depicted in Figure 11 together with the experimental results. Viscosity ratios of the four studied nanofluids at 313.15 K are plotted in the insert of Figure 11. NePCM viscosities rise with the concentration of graphene platelets up to 24%. The influence of material dispersion with different aspect ratios such as fibres or flakes can be described using the Maron and Piece model [69]:

(9) $$\frac{\eta_{\mathrm{nf}}}{\eta_{\mathrm{bf}}}={\left(1-\frac{\phi_{a}}{\phi_{m}}\right)}^{-2}$$

where *φ*_a_ is the effective volumetric fraction of aggregates, which reduces to the volume fraction of nanoplatelets in absence of aggregates and *φ*_m_ is the maximum packing volume fraction [70]. In this study, an AAD% of 1.4% was obtained using *φ*_m_ as adjustment parameter, obtaining an overall value of *φ*_m_ = 0.032 for the whole temperature range, which is about ten times larger than the highest volume fraction, for the whole temperature range. AAD% is reduced to 0.9% by considering temperature dependence of *φ*_m_, which takes values ranging from 0.030 to 0.051. The goodness of this model can be observed in the inset of Figure 11.

### 3.7. Evaluation Based on the Studied Properties

**Figure 12:**
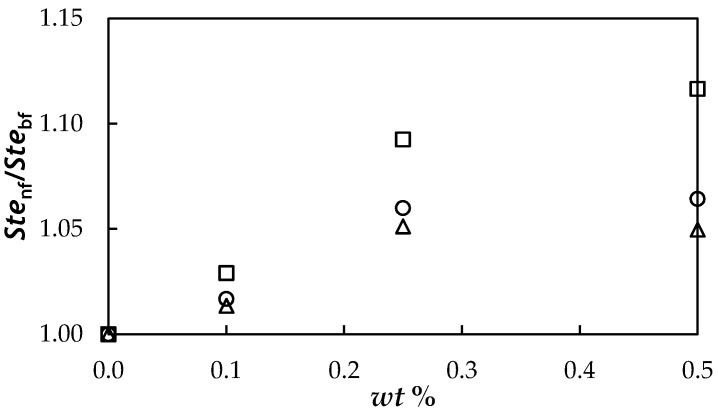
Concentration dependence of the ratio between Stefan numbers, *Ste*_nf_/*Ste*_bf_, at different temperatures: 293.15 K (□), 313.15 K (○) and 333.15 K (△).

With the aim of evaluating storage and heat transfer potentials for proposed NePCMs in relation to base material, PEG 400, the Figures of Merit (FOMs) known as Stefan and Rayleigh numbers were analysed. Stefan dimensionless number, *Ste*, represents the ratio between sensible heat, *C_p_*·*ΔT* and latent heat, *L_f_*, in the storage medium:

(10) $$Ste=\frac{C_{p}\cdot \Delta T}{L_{f}}$$

where *ΔT* = *T* − *T*_m_ is the difference between the temperature considered for the liquid phase, *T* and the melting temperature, *T*_m_. Figure 12 presents the concentration dependence of Stefan number at different temperatures. As stated above, no significant modification in the isobaric heat capacities was observed in comparison with the PEG 400 used as base material. However, decreases in temperature and enthalpy of melting transition with the addition of graphene nanoplatelets lead to a slight increase in Stefan number. This increasing trend with fGnP concentration is reduced when high liquid phase temperatures are considered.

**Figure 13:**
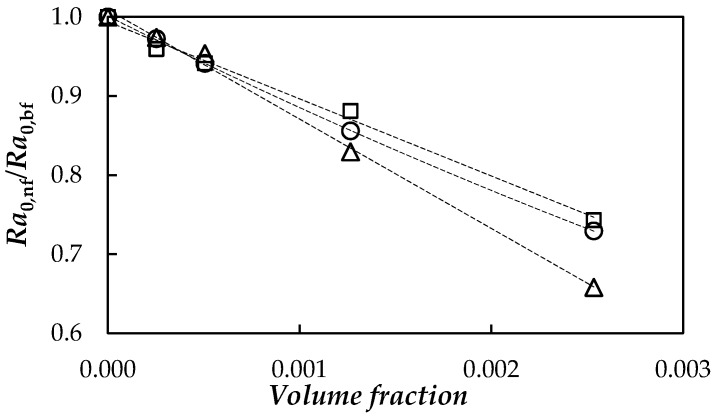
Rayleigh numbers ratio, *Ra*_0,nf_/*Ra*_0,bf_, vs. volume fraction at different temperatures: 293.15 K (□), 313.15 K (○) and 333.15 K (△).

Rayleigh number, *Ra*, represents the ratio between buoyancy force driving convection and viscous force resisting movement in the fluid and can be expressed in a dimensionless way, *Ra*_0_, as follows:

(11) $$Ra_{0}=\frac{Ra}{\Delta T\cdot L^{3}}=\frac{g\cdot \rho^{2}\cdot \alpha_{p}\cdot C_{p}}{\eta \cdot k}$$

where the temperature difference, *∆T* and length, *L*, correspond to the geometric and operating conditions of the system and *g* is the acceleration of gravity. Figure 13 shows the fGnPs concentration dependence of the ratio between the Rayleigh numbers, *Ra*_0_, obtained for each NePCM and base fluid.

As can be observed, Rayleigh number decreases with the dispersion of nanoplatelets due to the highest influence of the modifications in viscosity and thermal conductivity. A decrease in the Rayleigh number indicates a higher prevalence of the conduction processes in comparison to those of convection, which is more pronounced at higher temperatures.

## 4. Conclusions and Future Works

Four dispersions of functionalized graphene nanoplatelets up to 0.5 wt % in a poly(ethylene glycol), PEG 400, were designed and characterized for their possible use as NePCMs. Nanoplatelets present a rough laminar structure with thicknesses of several nanometres, while the poly(ethylene glycol) used as base PCM is a quasi monodisperse polymer with an mass-average molecular mass of 427.2 g·mol^−1^. According to the study of temporal dispersion stability, the optimal preparation conditions consist of a sonication treatment with an ultrasonic homogenizer for 70 min combined with mechanical stirring. Crystallization temperature decreases with the cooling rate for both PEG 400 and NePCMs. Nanoplatelet dispersion reduces crystallization temperature and the interval in which melting occurs up to in 4 K and 2.5 K, respectively. Good agreement was found between thermophysical property results here presented for the base fluid and those previously reported in literature. Thermal conductivity enhancements reach 23% for 0.50% fGnP/PEG 400 nanofluid. In relation to nanoparticle concentration dependence of isobaric heat capacity, density, thermal diffusivity and viscosity of NePCMs, we have found the characteristic behaviour in nanostructured thermal materials. Maximum modifications with respect to base fluid in *C_p_*, *ρ*, α and *ƞ* are 0.34%, 0.33%, 21% and 23%, respectively. Lower melting temperatures of NePCMs in comparison to PEG 400 leads to a slight increase in Stefan number, while Rayleigh number decreases with the dispersion of nanoplatelets due to viscosity and thermal conductivity increases. Other nanoadditives (such as metallic nanoparticles, different carbon nanostructures or other functionalizations) and/or poly(ethylene glycols) with different mass-average molar masses should be considered in future works in order to analyse the influence of nanoadditive nature and polymer chain length on PEG-based NePCMs.

## Figures and Tables

**Table 1:** Crystallization, *T*_crys_, and melting temperatures, *T*_m_, and latent heat of fusion, *ΔH*_m_, of PEG 400 and NePCMs obtained with heating and cooling rates of 1 K·min^−1^.

Nanoparticle Concentration, wt %	*T*_crys_ (K)	*T*_m_ (K)	*ΔH*_m_ (J·g^−1^)
PEG 400, base fluid 0%	271.6	278.8	105.3
fGnP/PEG400 NePCM, 0.10 wt %	269.9	278.5	104.4
fGnP/PEG400 NePCM, 0.25 wt %	268.3	277.7	100.7
fGnP/PEG400 NePCM, 0.50 wt %	267.6	276.3	97.2

## References

[1] Karaman S., Karaipekli A., Sarı A., Biçer A. (2011). Polyethylene glycol (PEG)/diatomite composite as a novel form-stable phase change material for thermal energy storage. Sol. Energy Mater. Sol. Cells.

[2] Pielichowska K., Pielichowski K. (2014). Phase change materials for thermal energy storage. Prog. Mater. Sci..

[3] Sharma A., Tyagi V.V., Chen C.R., Buddhi D. (2009). Review on thermal energy storage with phase change materials and applications. Renew. Sustain. Energy Rev..

[4] Cabeza L.F., Martorell I., Miró L., Fernández A.I., Barreneche C. (2015). Introduction to Thermal Energy Storage (TES) Systems.

[5] Sun Q., Yuan Y., Zhang H., Cao X., Sun L. (2017). Thermal properties of polyethylene glycol/carbon microsphere composite as a novel phase change material. J. Therm. Anal. Calorim..

[6] Qian T., Li J., Ma H., Yang J. (2015). The preparation of a green shape-stabilized composite phase change material of polyethylene glycol/SiO_2_ with enhanced thermal performance based on oil shale ash via temperature-assisted sol–gel method. Sol. Energy Mater. Sol. Cells.

[7] Tang B., Wu C., Qiu M., Zhang X., Zhang S. (2014). PEG/SiO_2_–Al_2_O_3_ hybrid form-stable phase change materials with enhanced thermal conductivity. Mater. Chem. Phys..

[8] Ma Z., Lin W., Sohel M.I. (2016). Nano-enhanced phase change materials for improved building performance. Renew. Sustain. Energy Rev..

[9] Gerard F., Camila B., Aran S., José Enrique J., Luisa F.C. (2017). Recent patents on nano-enhanced materials for use in thermal energy storage (TES). Recent Pat. Nanotechnol..

[10] Fandiño O., Lugo L., Comuñas M.J.P., López E.R., Fernández J. (2010). Temperature and pressure dependences of volumetric properties of two poly(propylene glycol) dimethyl ether lubricants. J. Chem. Thermodyn..

[11] Paredes X., Fandiño O., Pensado A.S., Comuñas M.J.P., Fernández J. (2012). Pressure-viscosity coefficients for polyalkylene glycol oils and other ester or ionic lubricants. Tribol. Lett..

[12] Zin V., Barison S., Agresti F., Colla L., Pagura C., Fabrizio M. (2016). Improved tribological and thermal properties of lubricants by graphene based nano-additives. RSC Adv..

[13] Gupta B., Kumar N., Panda K., Dash S., Tyagi A.K. (2016). Energy efficient reduced graphene oxide additives: Mechanism of effective lubrication and antiwear properties. Sci. Rep..

[14] Min X., Fang M., Huang Z., Liu Y., Huang Y., Wen R., Qian T., Wu X. (2015). Enhanced thermal properties of novel shape-stabilized PEG composite phase change materials with radial mesoporous silica sphere for thermal energy storage. Sci. Rep..

[15] Tang B., Cui J., Wang Y., Jia C., Zhang S. (2013). Facile synthesis and performances of PEG/SiO_2_ composite form-stable phase change materials. Sol. Energy.

[16] Wang P., Li N., Zhao C., Wu L., Han G. (2014). A phase change storage material that may be used in the fire resistance of building structure. Procedia Eng..

[17] Wang W., Yang X., Fang Y., Ding J. (2009). Preparation and performance of form-stable polyethylene glycol/silicon dioxide composites as solid–liquid phase change materials. Appl. Energy.

[18] Wang W., Yang X., Fang Y., Ding J., Yan J. (2009). Enhanced thermal conductivity and thermal performance of form-stable composite phase change materials by using β-aluminium nitride. Appl. Energy.

[19] Tang B., Qiu M., Zhang S. (2012). Thermal conductivity enhancement of PEG/SiO_2_ composite PCM by in situ Cu doping. Sol. Energy Mater. Sol. Cells.

[20] Tang B., Wei H., Zhao D., Zhang S. (2017). Light-heat conversion and thermal conductivity enhancement of PEG/SiO_2_ composite PCM by in situ Ti_4_O_7_ doping. Sol. Energy Mater. Sol. Cells.

[21] Zafarani-Moattar M.T., Majdan-Cegincara R. (2013). Investigation on stability and rheological properties of nanofluid of ZnO nanoparticles dispersed in poly(ethylene glycol). Fluid Phase Equilib..

[22] Zendehasbagh S., Majdan-Cegincara R. (2018). Magnetorheological and volumetric properties of starch and polyethylene glycol solutions in the presence of nio nanoparticles. Phys. Chem. Res..

[23] Amiri A., Sadri R., Shanbedi M., Ahmadi G., Chew B.T., Kazi S.N., Dahari M. (2015). Performance dependence of thermosyphon on the functionalization approaches: An experimental study on thermo-physical properties of graphene nanoplatelet-based water nanofluids. Energy Convers. Manag..

[24] Mehrali M., Sadeghinezhad E., Latibari S.T., Kazi S.N., Mehrali M., Zubir M.N.B.M., Metselaar H.S.C. (2014). Investigation of thermal conductivity and rheological properties of nanofluids containing graphene nanoplatelets. Nanoscale Res. Lett..

[25] Qi G.Q., Liang C.L., Bao R.Y., Liu Z.Y., Yang W., Xie B.H., Yang M.B. (2014). Polyethylene glycol based shape-stabilized phase change material for thermal energy storage with ultra-low content of graphene oxide. Sol. Energy Mater. Sol. Cells.

[26] Żyła G., Fal J., Estellé P. (2017). The influence of ash content on thermophysical properties of ethylene glycol based graphite/diamonds mixture nanofluids. Diam. Relat. Mater..

[27] Khodadadi J.M., Fan L., Babaei H. (2013). Thermal conductivity enhancement of nanostructure-based colloidal suspensions utilized as phase change materials for thermal energy storage: A review. Renew. Sustain. Energy Rev..

[28] Zhang Z., Fang X. (2006). Study on paraffin/expanded graphite composite phase change thermal energy storage material. Energy Convers. Manag..

[29] Sarı A., Karaipekli A. (2007). Thermal conductivity and latent heat thermal energy storage characteristics of paraffin/expanded graphite composite as phase change material. Appl. Therm. Eng..

[30] Kim S.D.L.T. (2009). High latent heat storage and high thermal conductive phase change materials using exfoliated graphite nanoplatelets. Sol. Energy Mater. Sol. Cells.

[31] Bahiraei F., Fartaj A., Nazri G.-A. (2017). Experimental and numerical investigation on the performance of carbon-based nanoenhanced phase change materials for thermal management applications. Energy Convers. Manag..

[32] Sari A., Karapekl A., Kaygusuz K. (2008). Fatty acid/expanded graphite composites as phase change material for latent heat thermal energy storage. Energy Sources Part A.

[33] Sarı A., Karaipekli A. (2009). Preparation, thermal properties and thermal reliability of palmitic acid/expanded graphite composite as form-stable PCM for thermal energy storage. Sol. Energy Mater. Sol. Cells.

[34] Karaipekli A., Sarı A., Kaygusuz K. (2007). Thermal conductivity improvement of stearic acid using expanded graphite and carbon fiber for energy storage applications. Renew. Energy.

[35] Li C., Xie B., Chen J. (2017). Graphene-decorated silica stabilized stearic acid as a thermal energy storage material. RSC Adv..

[36] Wang C., Wang W., Xin G., Li G., Zheng J., Tian W., Li X. (2016). Phase change behaviors of PEG on modified graphene oxide mediated by surface functional groups. Eur. Polym. J..

[37] Wang F., Zhang P., Mou Y., Kang M., Liu M., Song L., Lu A., Rong J. (2017). Synthesis of the polyethylene glycol solid-solid phase change materials with a functionalized graphene oxide for thermal energy storage. Polym. Test..

[38] Zhang H., Wu Q., Lin J., Chen J., Xu Z. (2010). Thermal conductivity of polyethylene glycol nanofluids containing carbon coated metal nanoparticles. J. Appl. Phys..

[39] Zhang X., Huang Z., Ma B., Wen R., Min X., Huang Y., Yin Z., Liu Y., Fang M., Wu X. (2016). Preparation and performance of novel form-stable composite phase change materials based on polyethylene glycol/white carbon black assisted by super-ultrasound-assisted. Thermochim. Acta.

[40] Wang Z., Zhang X., Jia S., Zhu Y., Chen L., Fu L. (2017). Influences of dynamic impregnating on morphologies and thermal properties of polyethylene glycol-based composite as shape-stabilized PCMs. J. Therm. Anal. Calorim..

[41] Liu Z., Wei H., Tang B., Xu S., Shufen Z. (2018). Novel light–driven CF/PEG/SiO_2_ composite phase change materials with high thermal conductivity. Sol. Energy Mater. Sol. Cells.

[42] Tang B., Wang Y., Qiu M., Zhang S. (2014). A full-band sunlight-driven carbon nanotube/PEG/SiO_2_ composites for solar energy storage. Sol. Energy Mater. Sol. Cells.

[43] Pop E., Varshney V., Roy A.K. (2012). Thermal properties of graphene: Fundamentals and applications. MRS Bull..

[44] Mehrali M., Tahan Latibari S., Mahlia T.M.I., Metselaar H.S.C., Naghavi M.S., Sadeghinezhad E., Akhiani A.R. (2013). Preparation and characterization of palmitic acid/graphene nanoplatelets composite with remarkable thermal conductivity as a novel shape-stabilized phase change material. Appl. Therm. Eng..

[45] Agromayor R., Cabaleiro D., Pardinas A.A., Vallejo J.P., Fernandez-Seara J., Lugo L. (2016). Heat transfer performance of functionalized graphene nanoplatelet aqueous nanofluids. Materials.

[46] Qian T., Li J., Feng W., Nian H.E. (2017). Single-walled carbon nanotube for shape stabilization and enhanced phase change heat transfer of polyethylene glycol phase change material. Energy Convers. Manag..

[47] Wang C., Feng L., Yang H., Xin G., Li W., Zheng J., Tian W., Li X. (2012). Graphene oxide stabilized polyethylene glycol for heat storage. Phys. Chem. Chem. Phys..

[48] Qian T., Li J., Ma H., Yang J. (2016). Adjustable thermal property of polyethylene glycol/diatomite shape-stabilized composite phase change material. Polym. Compos..

[49] Cabaleiro D., Gracia-Fernández C., Lugo L. (2014). (Solid + liquid) phase equilibria and heat capacity of (diphenyl ether + biphenyl) mixtures used as thermal energy storage materials. J. Chem. Thermodyn..

[50] Cabaleiro D., Colla L., Agresti F., Lugo L., Fedele L. (2015). Transport properties and heat transfer coefficients of ZnO/(ethylene glycol + water) nanofluids. Int. J. Heat Mass Transf..

[51] Cabaleiro D., Nimo J., Pastoriza-Gallego M.J., Piñeiro M.M., Legido J.L., Lugo L. (2015). Thermal conductivity of dry anatase and rutile nano-powders and ethylene and propylene glycol-based TiO_2_ nanofluids. J. Chem. Thermodyn..

[52] Gaciño F.M., Regueira T., Lugo L., Comuñas M.J.P., Fernández J. (2011). Influence of molecular structure on densities and viscosities of several ionic liquids. J. Chem. Eng. Data.

[53] Pielichowski K., Flejtuch K. (2002). Differential scanning calorimetry studies on poly(ethylene glycol) with different molecular weights for thermal energy storage materials. Polym. Adv. Technol..

[54] Wunderlich B. (1976). Macromolecular Physics: Crystal Nucleation, Growth, Annealing.

[55] Francesconi R., Bigi A., Rubini K., Comelli F. (2007). Molar heat capacities, densities, viscosities and refractive indices of poly(ethylene glycols) + 2-methyltetrahydrofuran at (293.15, 303.15 and 313.15) K. J. Chem. Eng. Data.

[56] O’Hanley H., Buongiorno J., McKrell T., Hu L.W. (2015). Measurement and model validation of nanofluid specific heat capacity with differential scanning calorimetry. Adv. Mech. Eng..

[57] Zhou S.Q., Ni R. (2008). Measurement of the specific heat capacity of water-based Al_2_O_3_ nanofluid. Appl. Phys. Lett..

[58] Nan C.W., Birringer R., Clarke D.R., Gleiter H. (1997). Effective thermal conductivity of particulate composites with interfacial thermal resistance. J. Appl. Phys..

[59] Kole M., Dey T.K. (2013). Investigation of thermal conductivity, viscosity and electrical conductivity of graphene based nanofluids. J. Appl. Phys..

[60] Cabaleiro D., Colla L., Barison S., Lugo L., Fedele L., Bobbo S. (2017). Heat transfer capability of (ethylene glycol + water)-based nanofluids containing graphene nanoplatelets: Design and thermophysical profile. Nanoscale Res. Lett..

[61] Afzal W., Mohammadi A.H., Richon D. (2009). Volumetric properties of mono-, di-, tri- and polyethylene glycol aqueous solutions from (273.15 to 363.15) K: Experimental measurements and correlations. J. Chem. Eng. Data.

[62] Niu Y., Gao F., Zhu R., Sun S., Wei X. (2013). Solubility of dilute SO_2_ in mixtures of n,n-dimethylformamide + polyethylene glycol 400 and the density and viscosity of the mixtures. J. Chem. Eng. Data.

[63] Živković N.V., Šerbanović S.S., Kijevčanin M.L., Živković E.M. (2013). Volumetric and viscometric behavior of binary systems 2-butanol + PEG 200, + PEG 400, + tetraethylene glycol dimethyl ether and + n-methyl-2-pyrrolidone. J. Chem. Eng. Data.

[64] Trivedi S., Pandey S. (2011). Densities of 1-butyl-3-methylimidazolium hexafluorophosphate + poly(ethylene glycol) in the temperature range (283.15 to 363.15) K. J. Chem. Eng. Data.

[65] Yoon D., Son Y.-W., Cheong H. (2011). Negative thermal expansion coefficient of graphene measured by raman spectroscopy. Nano Lett..

[66] Babapoor A., Karimi G. (2015). Thermal properties measurement and heat storage analysis of paraffinnanoparticles composites phase change material: Comparison and optimization. Appl. Therm. Eng..

[67] Cheng X., Li G., Yu G., Li Y., Han J. (2017). Effect of expanded graphite and carbon nanotubes on the thermal performance of stearic acid phase change materials. J. Mater. Sci..

[68] Bajić D.M., Ivaniš G.R., Visak Z.P., Živković E.M., Šerbanović S.P., Kijevčanin M.L. (2013). Densities, viscosities and refractive indices of the binary systems (PEG 200 + 1,2-propanediol, + 1,3-propanediol) and (PEG 400 + 1,2-propanediol, + 1,3-propanediol) at (288.15 to 333.15) K and atmospheric pressure: Measurements and modeling. J. Chem. Thermodyn..

[69] Maron S.H., Pierce P.E. (1956). Application of ree-eyring generalized flow theory to suspensions of spherical particles. J. Colloid Sci..

[70] Halelfadl S., Estellé P., Aladag B., Doner N., Maré T. (2013). Viscosity of carbon nanotubes water-based nanofluids: Influence of concentration and temperature. Int. J. Therm. Sci..

